# Radiographic and histological evidence of metabolic bone disease in gliding leaf frogs (*Agalychnis spurrelli*)

**DOI:** 10.1016/j.heliyon.2019.e01432

**Published:** 2019-04-05

**Authors:** María Cristina Galante-Mulki, Yessenia Alvear-Santos, Ana Cecilia Santamaría-Naranjo, Andrés Merino-Viteri, Alexander Genoy-Puerto

**Affiliations:** a Escuela de Medicina Veterinaria, Facultad de Ciencias de la Salud, Universidad de Las Américas, Colimes y Granados 170513, Ecuador; b Laboratorios Multidisciplinarios de Ciencias Biológicas y Químicas, Universidad de Las Américas, Colimes y Granados, Pichincha, Quito 170513, Ecuador; c Museo de Zoología (QCAZ), Escuela de Ciencias Biológicas, Pontificia Universidad Católica del Ecuador, Av. 12 de Octubre 1076 y Roca, Pichincha, Quito 170525, Ecuador

**Keywords:** Anatomy, Physiology, Ecology, Zoology

## Abstract

Bone alterations due to metabolic bone disease in captive animal populations can have a negative impact on repopulation and research initiatives. This investigation has the purpose of describing the principal radiographic and anatomopathological findings present in nine gliding leaf frogs (*Agalychnis spurrelli*) kept in captivity with alterations in their spines and long bones. The observed histopathological findings were in the canalis vertebralis, paraspinal muscle and long bones, and included deformed bones with alteration of the adjacent tissues, alterations in the ossification process, bone degeneration and resorption, decreased number of osteocytes and deposition of osteoid and fibrous material in the compact bone tissue. Additionally, the spinal cord showed compressed white matter, chronic meningitis in the duramater, alteration in the number of glial cells and loss of delimitation between the gray and white matter. Radiographical changes were found mainly in the long bones and included moth-eaten osteolysis, solid periosteal reaction, bone deformities, cortical tunneling and inflammation of adjacent soft tissues. Also, pathological fractures of the femur and urostyle were observed together with spinal column deviations with increased bone density.

## 1 Introduction

Disorders related to nutrition such as metabolic bone disease (MBD) or vitamin deficiencies are common in amphibians (Pessier et al., 2014). Adult and larval individuals are susceptible to mineral imbalances generating an abnormal calcium:phosphorus ratio (O'Rourke and Rosenbaum, 2015), inadequate levels of vitamin D_3_ and its biologically active form, calcitriol, and UV-B requirements (Antwis and Browne, 2009), or fluorosis (Shaw et al., 2012). In amphibians, the non-nutritional origin of MBD may be related to renal failure that would lead to secondary renal hyperparathyroidism (Pessier et al., 2014). The pathological presentation of metabolic bone disease is characterized by osteodystrophies, including rickets, osteomalacia, fibrous osteodystrophy and osteoporosis as individual presentations or in combination (Antwis and Browne, 2009). In the face of the severe extinction event in amphibians, it is recommended that the conservation community derive integrated and comprehensive strategies for effective conservation (Becker and Loyola, 2007). *Agalychnis spurrelli* is a nocturnal frog that inhabits humid forests located in Costa Rica, Panamá, Colombia and Ecuador in altitudes from 15 to 750 meters above the sea level (m.a.s.l.). It breeds in temporary rain pools through eggs that are laid on leaves. The conservation status of this species is currently classified by the International Union for Conservation of Nature (IUCN) Red List as Least Concern. However, this species may have conservation threats related to human pressure exerted by habitat loss and pollution (Ron et al., 2011), which may lead to the establishment of captive populations aiming for its conservation.

## 2 Materials and methods

The study was conducted according to the requirements for animal welfare accepted by the Universidad de Las Américas (UDLA) and Balsa de los Sapos Conservation Initiative (Pontificia Universidad Católica del Ecuador). The antemortem captivity and management conditions were approved and evaluated by the Ecuadorian Environmental Ministry (MAE) through the patent for wildlife management 2017 No 007-207-FAU-DPAP-MA.

Nine gliding leaf frogs, *Agalychnis spurrelli* (Hylidae) were used in this study: three females, three males, and three juveniles. All individuals were kept at “*Balsa de los Sapos”* Conservation Initiative of the Biological Sciences School at the *Potinficia Universidad Católica del Ecuador* in Quito, Ecuador. The specimens were maintained in glass terrariums with an environmental temperature that varied between 26 and 28 °C and a humidity that was around 60–80%. The individuals had exposure to UVB lights for 12 hours a day, although the people that worked in the conservation center did not measure the output at the level of the frogs. The substrate that they were on was composed by dried leafs and dirt that where collected from natural forests and autoclaved monthly. The water that they received was filtered with 20-inch thread, washable polyester, activated charcoal and exposed to UVB light, Hydrotech PURA ® filter. The water was never analyzed to determine the levels of minerals such as calcium or fluoride. The individuals were not genetically related.

The studied animals were wild caught from Esmeraldas, Ecuador (22.22% = 2/9) and born in the conservation centre (77.77% = 7/9). The animals were kept for 13 (66.66% = 6/9), 14 (22.22% = 2/9) and 15 (11.11% = 1/9) months until they were euthanized.

The animals were not swabbed for amphibian chytrid. The frogs were fed *ad libitum* mainly with an average of 10 farmed gut-loaded crickets dusted with vitamin and calcium supplements (Repashy Superfoods Calcium Plus®) 3 times a week, the average time of consumption was not determined. The crickets measured approximately 1 cm and were fed *ad libitum* with vegetables, rabbit and chicken food (WAYNE®).

The studied animals belonged to a population that showed signs of anorexia, lethargy, low body condition and limb weakness and displayed a spine deviation. Individuals had their mouth-cloaca distance measured by caliper (Scala®) and were weighed by digital balance (Boeco® BBI-41). Additionally, abdominal circumference was measured with a tape-measure.

After the initial evaluation, the animals were euthanized. The euthanasia protocols followed the Guidelines for the Euthanasia of Animals, of the American Association of Veterinary Medicine (2013), with topical benzocaine hydrochloride overdose (182 mg/kg). Full necropsies and the samples obtained were processed based on established protocols at the Histopathology Laboratory of Universidad de Las Américas ( Pessier and Pinkerton, 2003; Shilton et al., 2008).

Morphological analysis was made through biometric measures and radiological diagnosis. Radiographs were used to evaluate anatomical deformations. The X-rays images were acquired at the Biological Sciences School at the *Pontificia Universidad Católica del Ecuador* using the Thermo Kevex X-Ray PXS5 equipment (Glenbrook Technologies Inc, Randolph, NJ, UA). It was set at 40 kilovoltage (kV) and 0.1 milliamperage (mA), in order to take the radiographic plates. The animal was placed in ventral decubitus on an acetate sheet that was placed inside the X-ray chamber. The radiographic images were obtained by Image Acquisition VIVA™ software (Varian Medical Systems, Inc, Palo Alto, CA, USA).

Additionally, the lesions observed in the spinal column were classified in mild, moderate and severe depending on the number of vertebral bodies and intervertebral spaces that presented alterations as shown in Table 1.

**Table 1:** Classification system for the spinal column alterations.

Number of affected vertebral bodies and intervertebral spaces	Degree of affectation
One to two vertebral bodies and intervertebral spaces	Mild
Three to five vertebral bodies and intervertebral spaces	Moderate
More than five vertebral bodies and intervertebral spaces	Severe

Because alterations in long bones varied significantly between individuals, the lesions were considered for the study but could not be classified.

Tissues were sampled and collected in 10% buffered formalin. For histologic examination, the vertebral columns of all affected toads were decalcified (Kang et al., 2003). Transverse sections at intervertebral joints sections of the vertebral column were taken, embedded in paraffin, sectioned at 4 μm and stained with hematoxylin and eosin (HE) techniques. Additionally, formalin-fixed tissues, including heart, lung, liver, spleen, stomach, intestine, pancreas, kidney, bladder, gonad, skeletal muscle and skin, were processed as above, stained with HE, and microscopically examined.

## 3 Results

### 3.1 Measurements

The average snout-vent length was greater in females (48.83 ± 10.17 mm) than males (45.58 ± 3.77 mm) and juveniles (48.83 ± 10.17 mm). The average weight for males was 6.00 ± 1.87 g, 5.83 ± 3.02 g for females and 3.69 ± 0.88 g for juveniles. Abdominal circumference was wider in females (66.33 ± 40.38 mm) than the other groups: males (30.50 ± 8.23 mm) and juveniles (30.00 ± 10.00 mm).

### 3.2 Morphological alterations

#### 3.2.1 Gross anatomical observations

The results from the axial formula corresponded to the animal species (eight vertebrae in the presacral region, one sacral vertebra and one in the postsacral region and the urostyle). Also, two males (3773-6 and 3764) had cloacal prolapse and alterations in the kidney such as nephromegaly and pale coloration with whitish edges. Long bones appeared deformed with solid protrusions of bone tissue, especially in the femur, radio-ulna and tibiofibula. Also, the spinal column had an S-form deviation in its thoracolumbar region and collapsed paravertebral tissues with narrowed joints.

#### 3.2.2 Radiological findings

**Fig. 1:**
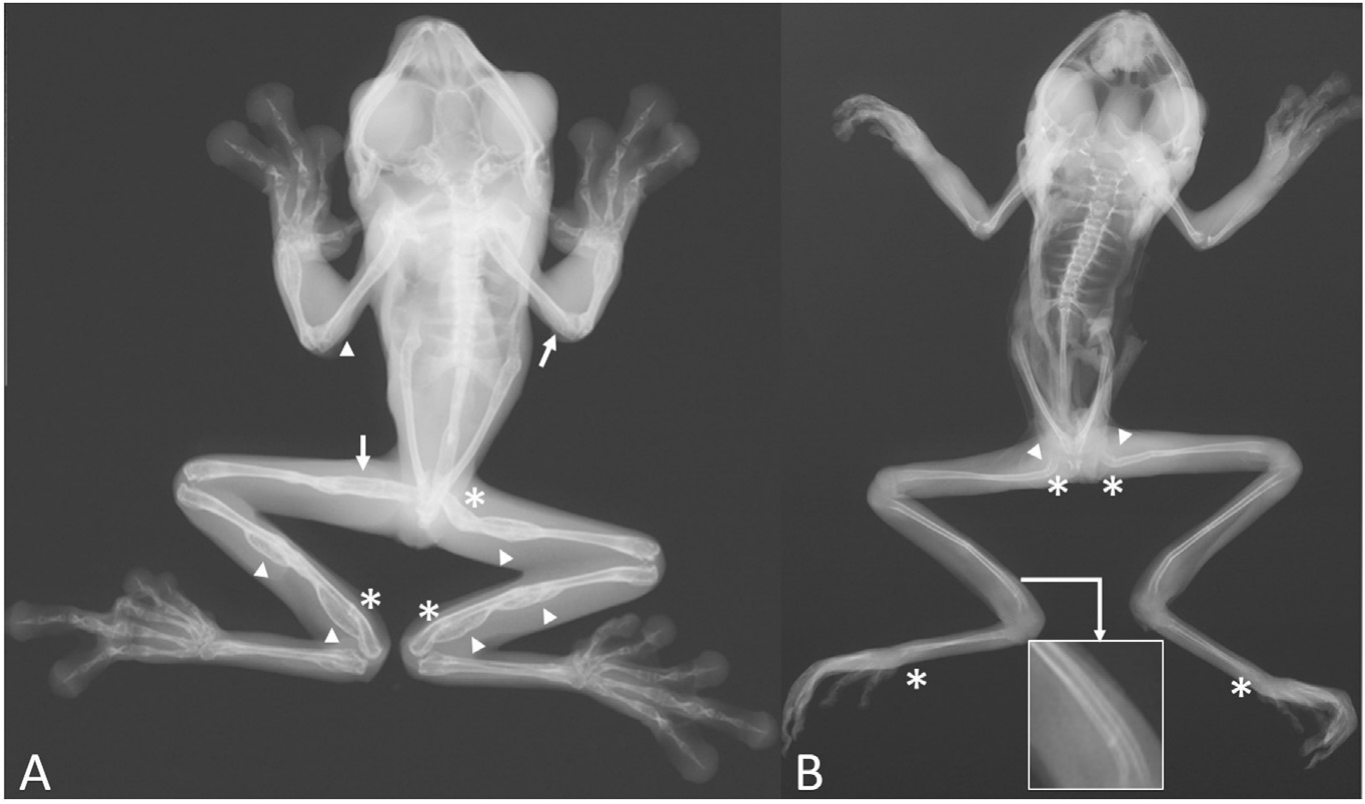
Bone alterations in X-ray images of the spinal column of gliding leaf frogs (*Agalychnis spurrelli*). (A) The individual presents moth-eaten osteolysis located in the distal epiphyses of the humerus and the proximal epiphysis of the femur (arrows), there are solid periosteal reactions in the distal epiphysis of both humerus and the spinal column presents increased bone density, the proximal epiphysis and diaphysis of the femur, the epiphysis and diaphysis of the tibiofibula (arrow heads). Also, in the proximal diaphysis of the femur and on the distal epiphysis of both tibiofibulas (asterisks) there is a bowing bone deformity (B) The proximal epiphysis of both femurs, and the distal epiphysis of both tibiofibulas, astragalus and calcaneus present a bowing bone deformity (asterisks), there is also mild solid periosteal reactions in the proximal epiphysis of both femurs and a lateral deviation of the lumbar section of the spinal column (arrow heads), the proximal and distal epiphysis of the tibiofibulas present cortical tunneling that becomes evident due to the presence of a radiolucent line in the middle of the cortical part of the bone (zoomed image indicated by the white arrow).

The radiological assessment showed moth-eaten osteolysis located in the epiphyses of the humerus and femur (11.11% = 1/9), increased spinal column bone density (22.22% = 2/9), solid periosteal reaction located mainly in the epiphysis of the humerus, femur, radio-ulna and astragalus and in the diaphysis of the femur and tibiofibula (66.66% = 6/9), pathological fractures located in the femur and urostyle (22.22% = 2/9), bone deformities located in the epiphysis of the femur, radio-ulna, tibiofibula, astragalus and calcaneus, and in the diaphysis of the femur, astragalus and calcaneus (88.88% = 8/9) and cortical tunneling located in the epiphysis of the tibiofibula, astragalus and calcaneus (33.33% = 3/10). In most cases, the alterations presented previously were joined by inflammation of soft tissues (77,77% = 7/9). These alterations are shown in Fig. 1. As for the spinal column deviations, half of the individuals (55.55% = 5/9) had a moderate condition, and (44.44% = 4/9) had mild involvement in the shape and direction of the vertebrae. This is summarized in Table 2.

**Table 2:** Effects on vertebra of gliding leaf frogs (*Agalychnis spurrelli*) impacted by bone malformations in the spinal column.

Identification	Affected vertebra
3764	Urostyle deviation
3773(2)	Deviation in 7^th^ vertebra and sacral vertebra
3773(3)	Deviation in 2^nd^ and 3^th^ vertebrae and increase in intervertebral space in the same section
3773(4)	Deviation in 6^th^ and 7^th^ vertebrae
3773(5)	Deviation in 5^th^, 6^th^, and 7^th^ vertebrae, sacral vertebra and urostyle
3773(6)	Deviation in 6^th^ and 7^th^ vertebrae. Fracture of urostyle
3773(7)	Deviation in 4^th^ and 5^th^ vertebrae. Decrease in intervertebral space 3^rd^ - 4^th^
3774	Mild deviation in 7^th^ and 8^th^ vertebrae and urostyle
3777	Deviation in 5^th^ and 6^th^ vertebrae. Increase in intervertebral space 7^th^-8^th^. Deviation of urostyle

### 3.3 Histopathological findings

#### 3.3.1 Canalis vertebralis

**Fig. 2:**
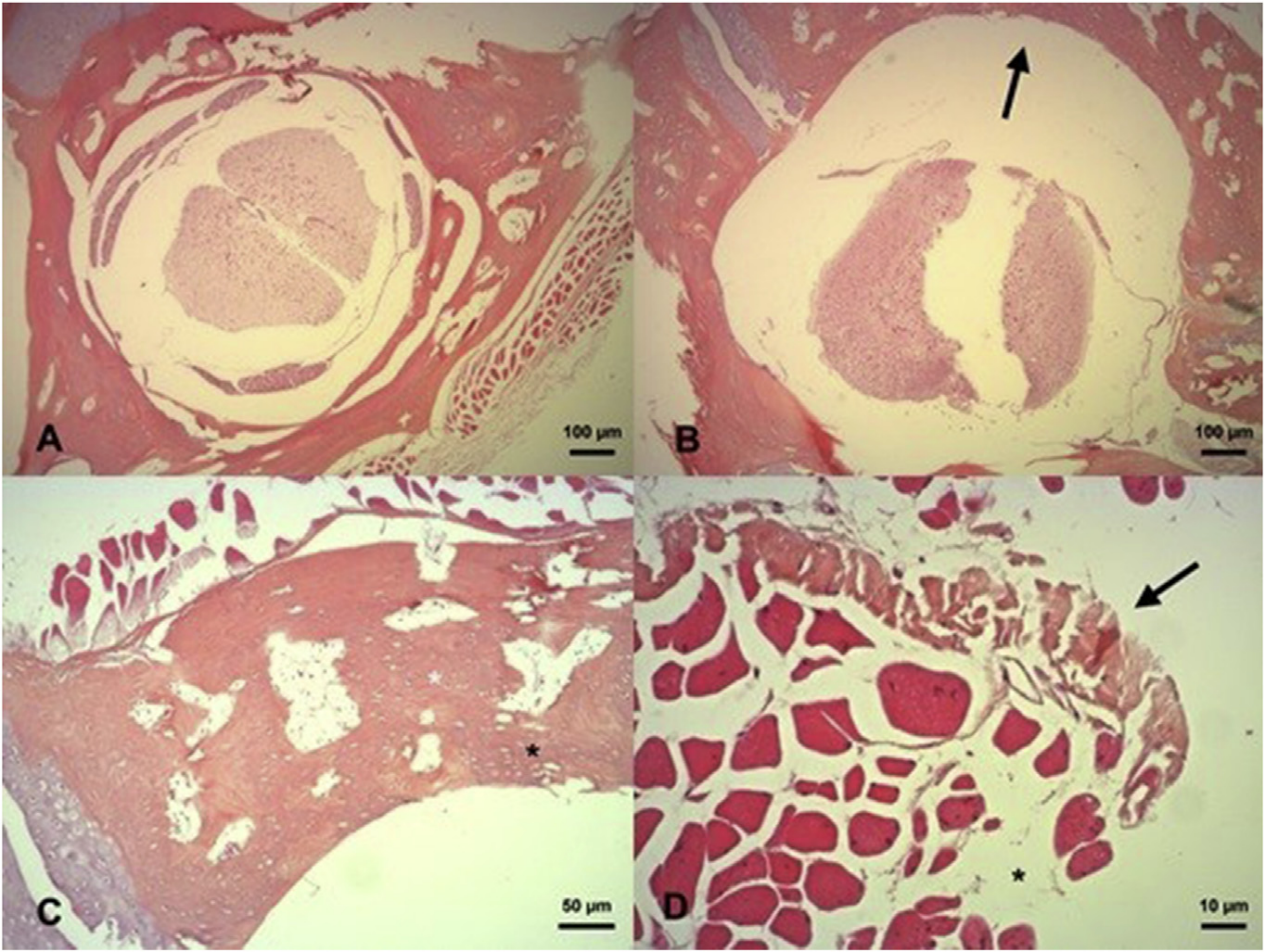
Photomicrographs of transversal sections of vertebral bodies and paraspinal muscle of gliding leaf frogs (*Agalychnis spurrelli*). (A) Vertebral foramen without lateral apertures. (B) Increased size of the vertebral foramen with lateral acute aperture (black arrow). (C) Altered paraspinal muscle with pale (black arrow) and hypereosinophilic areas, loss of muscle fibers and increased size of endomysium (asterisk). (D) Areas of degeneration with a wider gap between the osteocytes and the lacunae (black asterisk). The size of the osteocytes was also reduced and the bone canaliculus diminished (white asterisk).

Because a single individual may have several alterations in the same bone as can be observed in Fig. 2, the percentages were calculated for each alteration*.* In (66.70% = 6/9) of the individuals, the vertebral foramen was round with acute apertures (33.30% = 3/9) had an ovoid-shaped foramen; and (44.00% = 4/9) exhibited a sharp prolongation that compressed the paraspinal muscle.

Because normal values in the species haven't been determined, in other alterations, the comparison was made between the analyzed individuals, taking into consideration that the normal canalis vertebralis should not contain cartilage in adult individuals and should present an elliptic aperture for the spinal cord (Liu et al., 2016).

Thus, the size of the vertebral foramen was increased in (44.00% = 4/9) of the animals and decreased in (11.00% = 1/9) in relation to the average vertebral foramen observed in comparison to the other frogs. Additionally, the presence of cartilage was evident in the vertebral processes (88.90% = 8/9) of the studied amphibians. Another important finding was the presence of degenerated areas with bone marrow exposure, which was surrounded by altered vertebral tissue with enlarged lacunae. In (88.90% = 8/9) of the individuals, these changes were accompanied by a decreased number of osteocytes. The bone canaliculi were increased in size and number in (22.20% = 2/9), while in (77.80% = 7/9), they decreased in comparison to the average bone canaliculi observed in the rest of the population.

#### 3.3.2 Spinal cord

The spinal cord of (88.90% = 8/9) of the individuals exhibited compressed white matter, which was accompanied by compression of the spinal nerves in (22,2% = 2/9) cases and by chronic meningitis located in the duramater in (11.11% = 1/9) cases. Another common change observed was the presence of focal congestion in (67.00% = 6/9). Pathological alterations were also observed in the number of non-ependymal glial cells, which was reduced in (55.6% = 5/9) and increased in (22.2% = 2/9) of the individuals. For the ependymal glial cells, their number was increased in (69.00% = 6/9) of the observed anurans. This change was accompanied by loss of delimitation between the gray and white matter (44.00% = 4/9) and by a focal increase in the cell nuclear size in (33.30% = 3/9) of the individuals. The central canal of the spinal cord also showed alterations of its size, which was reduced in (33.30% = 3/9) of the cases and expanded in (11.10% = 1/9).

#### 3.3.3 Paraspinal muscle

The muscle presented alterations associated with necrosis and degeneration in all the studied individuals. In (88.90% = 8/9) of the cases, this change was observed altogether with a decreased number of fibers in the muscle fascicle and a focal enlargement of the endomysium. Another important alteration was morphological changes in the diameter of the muscle fibers that were reduced due to compression in (66.70% = 6/9). Changes in the disposition of myocytes in the muscle fibers were not common in this population, as only (11.11% = 1/9) cases exhibited agglomeration of myocytes and (11.11% = 1/9) cases exhibited muscular regeneration. In normal specimens the paraspinal muscle presented a uniform number and disposition of myocytes with peripheral nuclei.

#### 3.3.4 Long bone anomalies

**Fig. 3:**
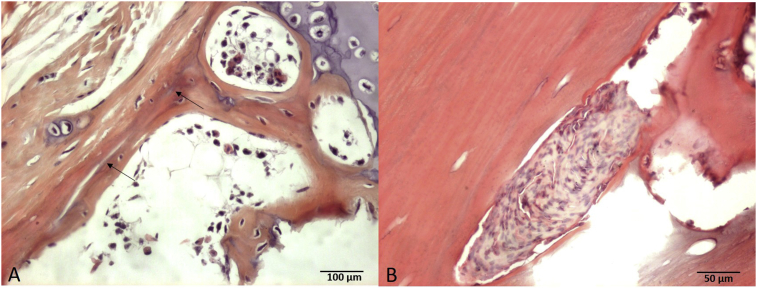
Photomicrographs of long bones of gliding leaf frogs (*Agalychnis spurrelli*). (A) Phalange with alteration of the subchondral bone, there are reversal lines (arrows) and active osteoclasis (asterisk). (B) Left femur with severe accumulation of fibrous material located inside the compact bone and active osteoclasis.

The main anomalies were located in the metatarsa, phalanges, femur, radio-ulna and tibiofibula. In the metatarsa and phalanges were Howship's lacunae with active osteoclasis surrounded by reversal lines joined by deposition of osteoid material in the subchondral bone, these pathological findings are compatible with osteomalacia (66.66% = 6/9). In the femur, radio-ulna and tibiofibula there were similar alterations but there was also fibrous material in the compact bone tissue, this alteration is compatible with fibrous osteodystrophy (33.33% = 3/9). No anomalies compatible with rickets were found in the cartilage of the growing plate. The alterations described previously can be observed in Fig. 3.

#### 3.3.5 Kidneys

The alterations found in the kidneys were tubular necrosis, inflammatory cell infiltration, edema and dilation of glomerular capillaries.

## 4 Discussion

### 4.1 Measurements

Snout-vent length in adult captive *Agalychnis spurrelli* frogs has been previously studied by other authors who reported that males can reach a size from 47.0 to 75.6 mm with an average of 65.90 mm, and females can have an average size of 76.4 mm with a range between 62.0–92.8 mm. In our study, the analyzed individuals had smaller snout-vent measurements; this difference could be related to pathological changes such as MBD. The relation between MBD and insufficient exposure to UVB rays has been proved in a prior study that observed that captive Amazonian milk frogs (*Trachycephalus resinifictrix*) that were exposed to UVB rays (>50 μw cm^−2^) for 30 minutes every two weeks had optimal growth and skeletal development (Verschooren et al., 2011).

### 4.2 Morphological changes

The most evident pathological alterations were observed in the canalis vertebralis, in the spinal cord, in the paraspinal muscles and in long bones.

#### 4.2.1 Necropsy

In amphibians the spine structures are prominent, the presacral region has no more than nine units, followed by a sacral vertebra and finally the urostyle in the postsacral region (Handrigan and Wassersug, 2007). This axial formula was very close to the one observed in the frogs evaluated in this study with the addition of the prominent spinal deviation. Similar morphological changes were observed in the body-curved Chinese giant salamanders included affected animals exhibiting ¨S¨ or ¨L¨ body shapes (scoliosis) (Liu et al., 2016). These alterations are similar to malformations in amphibians that are represented as inflammatory and degenerative conditions and even necrosis of vertebra and cartilage. This type of injury is also reported in the spinal cord and paraspinal muscles. The causes are associated with infections by *Ribeiroia ondatrae*, *Clinostomun* spp. *Ochrobactrum anthropi* (Johnson et al., 2002, Shilton et al., 2008) or low levels of calcium and vitamin D_3_ (Liu et al., 2016). No parasites or lesions compatible with parasite infections were found in this study.

The findings observed in long bones have been previously reported in domestic animals with elevation of PTH (parathyroid hormone), chronic renal disease and nutritional imbalances and also in reptiles and New World primates, in which the bone presents exuberant proliferation of fibrous tissue, making the bone appear enlarged and weakened (Uhl, 2018; Zachary and McGavin, 2012). In the present study the main alterations were found in bones that are generally exposed to mechanical stress such as the femur, radio-ulna and tibiofibula, which are bones that according to several authors are commonly affected by MBD (Craig et al., 2007; Klaphake, 2010). However, no alterations were found in the mandibular bone, which is a common alteration in reptiles and mammals affected by MBD (Zachary and McGavin, 2012).

The enlargement found in the kidneys have been described previously in cases of renal congestion, fat, edema and accumulation of urine. These alterations are associated with acute renal inflammation in which the kidney becomes bigger due to the addition of cells and fluid (Cianciolo and Mohr, 2016). Acute renal inflammation can have many different origins, the most common ones are decreased renal perfusion, ischemic tubular damage, decreased glomerular filtration and toxic tubular damage, tubulointerstitial inflammation and edema (Zachary and McGavin, 2012). In the present study, these alteration may have been originated by a lower renal perfusion due to the use of benzocaine during the euthanasia (Guertler et al., 1992). Benzocaine has been widely known to cause acute renal inflammation due to lower perfusion caused by methemoglobinemia (Guertler et al., 1992; Tilley and Smith, 2011). Other findings observed in the kidneys that support the idea of a lower perfusion were the presence of whitish edges and pale organ edges, anomalies that have been reported in cases of renal infarction (Tilley and Smith, 2011). Even though MBD have also been associated to renal failure, it is usually a consequence of a chronic process rather than an acute one. The fact that no chronic lesions such as parenchymal scarring with a reduced kidney size, suggests that the findings were not associated (Cianciolo and Mohr, 2016).

#### 4.2.2 Radiological findings

Metabolic bone disease is a common alteration that can be found in amphibians and that represents a diagnostic challenge. In most cases X-rays are the first tool that veterinarians and researchers use for approaching a correct diagnosis. However, the evaluation of radiographs can be complicated and nonspecific, due to the scarce information available about the radiological anomalies associated to MBD in amphibians. Luckily, in other species such as humans, mammals and reptiles the alterations have been studied deeply enough to let the professionals extrapolate them to give a proper diagnosis (Klaphake, 2010).

In the present study the radiological findings included increased spinal column bone density (22.22% = 2/9), epiphysial moth-eaten osteolysis (11.11% = 1/9), solid periosteal reaction located the epiphysis and diaphysis (66.66% = 6/9), bone deformities located in epiphysis and in the diaphysis (88.88% = 8/9), epiphysial cortical tunneling (33.33% = 3/10) and inflammation of soft tissues (77,77% = 7/9), pathological fractures of the femur and urostyle (22.22% = 2/9) and spinal column deviations (100% = 9/9). All these alterations have been described in previous studies in other species with metabolic bone disease (Chang et al., 2016; Freedman et al., 1976; Patel et al., 2015).

#### 4.2.3 Findings associated to osteomalacia and rickets

Osteomalacia is an anomaly that generates radiological evidence due to the pathological alterations that it generates in the mineralization process of the bone, whereas rickets is a defective mineralization of growth plates (Chang et al., 2016). In humans the most common radiographic findings that are present in affected patients are decreased bone density, looser zones, loss of cortical definition and bone deformities in weight-bearing bones (Chang et al., 2016; Patel et al., 2015). Even though, in the present study there were insufficient radiographic alterations related to decreased bone density, looser zones and loss of cortical definition, there were plenty of bone deformities located in mainly in the hindlimbs and the tibiafibula. Similar findings have also been observed in captive New Zealand Native Frogs (*Leiopelma* species) with MBD originated by fluorosis, which also presented bone deformities without a significant loss of bone radiopacity (Shaw et al., 2012).

#### 4.2.4 Findings associated to fibrous osteodystrophy

Even though fibrous osteodystrophy can't be diagnosed only by radiographic interpretation due to the problems described previously and to the fact that in many cases there may be more than one type of MBD in the same patient, there are a few radiographic findings that appear frequently in patients with this bone alteration (Chang et al., 2016; Zachary and McGavin, 2012). In humans, frequently described anomalies are subperiosteal bone resorption, periosteal reaction, intracortical, subchondral and endosteal resorption, brown tumors, pathologic fractures, diffuse increase in bone radiodensity and striped appearance of the spinal column (Chang et al., 2016; Patel et al., 2015). In reptiles there may be other additional anomalies such as a deformed spine and extensive periosteal bone formation that deforms the bone in an evident manner (Freedman et al., 1976). In this study, there were many alterations that were very similar, such as spinal column deformities, solid periosteal reaction in long bones, pathological fractures and epiphyseal cortical resorption or tunneling. Nevertheless, there were no evident signs of brown tumors, striped appearance of the spine and generalized increase in bone radiodensity (this alteration was only present in the spinal column). These changes in the presentation of the disease may suggest that MBD can react differently depending on the specie and type of bone that is affected.

These differences in the disease presentation and the need of further evidence make the performance of additional diagnostic techniques necessary. Diagnostic procedures that are more specific for the diagnosis of MBD include dual energy X-ray absorptiometry (DXA) that can help to quantify the bone density that the patient has. This value can be compared later with the reference body density to detect any pathological changes (Klaphake, 2010). Another method that can be used is microcomputer tomography scans (Micro CT) which provides a more detailed image of the bone, its deformations and alterations. This exam can be complemented with the usage of contrast agents that detect even bone microdamages (Boerckel et al., 2014; Shaw et al., 2012).

### 4.3 Histopathological findings

Bone alterations are usually associated with genetic and congenital diseases, nutritional and hormonal imbalances, intoxication, inflammatory and infectious bone diseases, tumors and tumor-like lesions. Of all the causes mentioned previously the most common ones are those related to MBD. Even though all of the MBD share similar characteristics and regardless of the fact that the same bone may present more than one of these abnormalities at the same time, there are certain pathological findings that may indicate the presence of a specific alteration. In this study, the canalis vertebralis of the analyzed individuals presented morphological changes in the bone structure and tissue, as well as abnormal ossification. These alterations are compatible with the ones that are regularly observed in cases of fibrous osteodystrophy and rickets, which are characterized by a defective bone formation with an altered mineralization that generates an inadequate process of ossification (Craig et al., 2007). Both rickets and fibrous osteodystrophy are a result of metabolic and hormonal imbalances due to various causes the most common ones are dietary deficiencies of vitamin D_3_, calcium and phosphorus; high dietary levels of iron; renal failure; or a combination of all of the above (Craig et al., 2007; Shaw et al., 2012). In anurans, the presence of MBD have been previously reported by several authors (Antwis and Browne, 2009; Klaphake, 2010; O'Rourke and Rosenbaum, 2015; Wright and Whitaker, 2001) who have defined that MBD can have many possible causes, among them are lack of exposure to UVB rays; low consumption of the vitamin D3 in the diet; digestive disorders that complicate the absorption of the vitamin such as pathologies of the small intestine, hepatobiliary system or pancreas; and lack of fat in the diet (Antwis and Browne, 2009; Craig et al., 2007). Even though all of the previously mentioned causes are commonly presented in captive amphibians, there is very limited information about the spinal column deformities that MBD can cause (Lannoo, 2008; Wright and Whitaker, 2001), which is why these lesions can only be compared to the ones found in other studies about spinal column malformations in adult individuals. In these other studies, the most common results were scoliosis (Liu et al., 2016; Schoff et al., 2003), spinal fractures (Shaw et al., 2012), sharp and collapsed canalis vertebralis with bigger lacunae and a lower number of osteocytes, and bone canaliculus (Liu et al., 2016). In the individuals analyzed in this study, we did find one case of bone fracture and similar morphological alterations in the canalis vertebralis and the bone tissue.

#### 4.3.1 Alterations in the spinal cord

In a study that analyzed spinal column changes in malformed breeding giant salamanders, the authors described that the most common alterations in the spinal cord were deformation of the central canal, increase in the size of glial cells and presence of ﬁbrous connective tissue (Liu et al., 2016). Even though we did not observe fibrous connective tissue in the spinal cord in this study, all of the other findings mentioned above are very similar to the ones found in the individuals analyzed in this research, particularly changes in the central canal and glial cells. Other pathological changes that we observed in this study were loss of delimitation between the gray and white matter, hydromyelia, congestion and alterations in the number of glial cells. All of these additional changes are very similar to the pathological findings that are present in cases of spinal cord compression in which the consequential congestion produces myelopathy with an abnormal cell morphology and disposition within the spinal cord (Craig et al., 2007; Cheriyan et al., 2014; Oldfield, 1992).

#### 4.3.2 Paraspinal muscle alterations

While muscles have been widely studied in veterinary medicine (Craig et al., 2007), paraspinal muscle lesions in amphibians with spinal cord alterations have been described by a very limited number of authors, who have identified that the most important lesions in the paraspinal muscles of these individuals are muscular deformation, shortening of the muscle fiber diameter and of the endomysium size with an increased number of nuclei in the myocytes (Liu et al., 2016). While in this study we observed a decreased size in the muscle fibers compatible with muscle atrophy (Craig et al., 2007), the pathological findings reported in the study mentioned previously are very different from the ones found in our study in which we observed focal pale and hypereosinophilic areas, focal enlargement of the endomysium, and a decreased number of muscle fibers. All of the pathological changes observed could have originated from necrosis and myodegeneration, which are characterized by the presence of fibrosis and cellular alterations with consequent staining abnormalities (Craig et al., 2007; Zachary and McGavin, 2012).

Together with the radiographic and histopathological lesions described here are compatible with a diagnosis of MBD. The husbandry provided was likely inadequate in providing a proper Ca:P ratio and/or a sufficient amount of UVB radiation. Several recommendations for captive populations are described in the literature. With some species in conservation breeding programs, the positive metabolic effects of UV-B in producing vitamin D_3_, or the provision of vitamin D_3_ through diet, may be necessary to maintain health. Consequently, a primary challenge in captive amphibians is the provision of adequate levels of either dietary vitamin D_3_ or UV-B lighting to enable synthesis of calcitriol in the interest of maintaining healthy populations (Antwis and Browne, 2009). Appropriate calcium supplementation may include administration of calcium carbonate on the food and gut loading the invertebrates (O'Rourke and Rosenbaum, 2015). Monitoring water quality is necessary to measure a wide range of minerals such as calcium, chloride, fluoride, magnesium, copper, iron, and lead (Shaw et al., 2012). UV-B radiation and dietary cholecalciferol can increase the skin-generated vitamin D_3_ synthesis (Klaphake, 2010).

#### 4.3.3 Long bone changes

Osteomalacia is defined as a defective mineralization process that creates deformities in adult bones. Rickets is a very similar alteration that affects the growth plate in young individuals. In the present study there were changes that could be associated with altered bone formation and mineralization such as reversal lines. Reversal lines appear as a result of a temporary interruption of bone resorption followed by formation, and show a disorganized modeling and remodeling (Zachary and McGavin, 2012). Another pathological finding that suggests that there were alterations in the bone tissue are Howship's lacunae, these cavities appear as a result of bone resorption and erosion created by osteoclasts, which are activated by calcitonin secretion. Together, these alterations suggest that there was an alteration in calcium metabolism that produced cyclic bone resorption periods (Zachary and McGavin, 2012). When there is a pathology such as MBD, the remodeling process is abnormal, and instead of forming new normal bone, there is a deposition of osteoid, like the one that was observed in the metatarsa or proliferation of fibrous tissue like the one observed in the femur, radio-ulna an tibiofibula (Craig et al., 2007). The bone deposition of osteoid is a characteristic that has been reported in cases of osteomalacia, and the formation of fibrous tissue is a representative finding of fibrous osteodystrophy, diseases that have been widely reported in reptiles, domestic and wildlife mammals and humans (Craig et al., 2007; Chang et al., 2016; Freedman et al., 1976; Klaphake, 2010; Patel et al., 2015; Zachary and McGavin, 2012).

#### 4.3.4 Kidney alterations

The alterations found in the kidney are widely unspecific, but according to several authors they may be a result of a shock state, dehydration or heart failure (Cianciolo and Mohr, 2016; Zachary and McGavin, 2012). Considering that no significant findings were observed in the heart of the studied individuals and that none of them showed evident signs of dehydration, the most probable cause of these alterations was shock. Since the studied individuals were euthanized it is highly possible that the findings were associated to the usage of benzocaine. Benzocaine is an anesthetic drug that induces methemoglobinemia, which consists in the oxidation of ferrous heme iron, causing it to lose its ability to carry oxygen (Guertler et al., 1992). The lack of oxygen can create a state of hypoxemia, which induces the organism to start a vascular mechanism that has the purpose of diverting blood to the most vital and essential organs. If the situation is not reversed the severe lack of oxygen will generate shock and finally death of the affected individual (Cianciolo and Mohr, 2016). Similar findings to the ones presented in this research have been reported in swine used in an experiment that evaluated the kidney damage present after asphyxiation and cardiac arrest, a situation that also exposes animals to severe hypoxia (Hang et al., 2014).

In conclusion, the radiographical findings together with the histopathological changes suggested that the individuals had an MBD, probably due to a vitamin D3 deficiency or an inadequate Ca:P correlation. Although, this study did not confirm the cause of these alterations it could guide the conservation center “Balsa de los Sapos” and other *Agalychnis spurrelli* breeding centres to improve the environmental and nutritional conditions provided to these animals.

## Declarations

### Author contribution statement

María Cristina Galante-Mulki, Yessenia Alvear-Santos, Ana Cecilia Santamaría-Naranjo, Andrés Merino-Viteri, Alexander Genoy-Puerto: Conceived and designed the experiments; Performed the experiments; Analyzed and interpreted the data; Contributed reagents, materials, analysis tools or data; Wrote the paper.

### Funding statement

This work was supported by the Directorate-General for Research of Universidad de Las Américas (UDLA), Ecuador (grant number VET. AG.17.04), and the General Academic Direction at Pontificia Universidad Católica del Ecuador for funding resources through K13039 and L13039 research projects.

### Competing interest statement

The authors declare no conflict of interest.

### Additional information

No additional information is available for this paper.
